# Support Vector Regression for Mobile Target Localization in Indoor Environments

**DOI:** 10.3390/s22010358

**Published:** 2022-01-04

**Authors:** Satish R. Jondhale, Vijay Mohan, Bharat Bhushan Sharma, Jaime Lloret, Shashikant V. Athawale

**Affiliations:** 1 Department of Electronics and Telecommunication, Amrutvahini College of Engineering, Sangamner 422608, Maharashtra, India; profsatishjondhale@gmail.com; 2 Department of Mechatronics, Manipal Institute of Technology, Manipal Academy of Higher Education, Manipal 576104, Karnataka, India; vijay13787@gmail.com; 3 School of Automation, Banasthali Vidyapith, Tonk 304022, Rajasthan, India; mbbs.sharma@gmail.com; 4 Instituto de Investigacion para la Gestion Integrada de Zonas Costeras, Universitat Politecnica de Valencia, Grao de Gandia, 46730 Valencia, Spain; 5 Department of Computer Engineering, AISSM College of Engineering, Pune 411001, Maharashtra, India; svathawale@gmail.com

**Keywords:** trilateration, received signal strength (RSS), wireless sensor network (WSN), localization and tracking (L&T), support vector regression (SVR), Kalman filter (KF), generalized regression neural network (GRNN)

## Abstract

Trilateration-based target localization using received signal strength (RSS) in a wireless sensor network (WSN) generally yields inaccurate location estimates due to high fluctuations in RSS measurements in indoor environments. Improving the localization accuracy in RSS-based systems has long been the focus of a substantial amount of research. This paper proposes two range-free algorithms based on RSS measurements, namely support vector regression (SVR) and SVR + Kalman filter (KF). Unlike trilateration, the proposed SVR-based localization scheme can directly estimate target locations using field measurements without relying on the computation of distances. Unlike other state-of-the-art localization and tracking (L&T) schemes such as the generalized regression neural network (GRNN), SVR localization architecture needs only three RSS measurements to locate a mobile target. Furthermore, the SVR based localization scheme was fused with a KF in order to gain further refinement in target location estimates. Rigorous simulations were carried out to test the localization efficacy of the proposed algorithms for noisy radio frequency (RF) channels and a dynamic target motion model. Benefiting from the good generalization ability of SVR, simulation results showed that the presented SVR-based localization algorithms demonstrate superior performance compared to trilateration- and GRNN-based localization schemes in terms of indoor localization performance.

## 1. Introduction

Target localization has been widely researched in recent years, especially to meet the demands of location based services (LBS) for various applications [1,2,3]. A number of examples of LBS can be given that are useful for uplifting the living standards of society. For instance, in a bike sharing service, a rider can rent a bike using a mobile app and drop it anywhere for the next user once the purpose of hiring is over. The exact locations of all available shared bikes are needed by interested riders in order to look for the nearest bike. Wearable devices such as smart watches can provide their owners with services such as activity monitoring, tracking, and emergency messages. In the retail industry, localization can help raise profits by finding customer locations and even guide them to specific products of interest. This certainly results in an improved shopping experience from the customer’s point of view and a gain in revenue from the owner’s point of view. One more very interesting example of LBS is location-based flow management (LBFM). In LBFM, location data of people in public spaces such as metros, airports, and railway stations can be utilized to study statistics on passengers, optimize their organization and provide necessary signaling to them. In industry, logistics, productivity and safety can easily be improved using the concept of LBS. A global positioning system (GPS) is a very popular technological option for location estimation of outdoor objects; however, the location estimates obtained with a GPS are not reliable and accurate in case of an indoor environment, due to unavailability of indoor GPS signals [4,5]. Therefore, GPS-less localization and tracking (L&T) systems are a must for indoor environments to obtain high target localization accuracy. Being a dominant wireless communication technology for the last three decades, a WSN can easily replace a GPS for indoor localization applications due to low cost, low power consumption and capabilities of smart sensing and ubiquitous computing [6,7,8]. 

Signal propagation in a wireless medium between a transmitter and a receiver basically involves location-dependent information, which can be utilized to locate the target of interest. This location-dependent information can be extracted from signal measurement metrics such as RSS, time of arrival (TOA), time difference of arrival (TDoA), angle of arrival (AOA), or combinations thereof [9]. Out of all these metrics, the RSS-based approach is the most preferred in WSN-based L&T, as unlike others, RSS-based localization systems do not involve the requirement of any additional hardware with the sensor node [10]. The dominant techniques for localization are range-based localization and range-free localization. In the range-based localization approach, the distance between a transmitter and a receiver is computed, whereas in range-free localization the distances are not computed. RSS has been used in both approaches. However, received signal strength indicator (RSSI) measurements are generally noisy and are of a highly fluctuating nature due to the complex radio frequency (RF) environment in indoor areas [11,12]. RSSI measurements suffer from diverse indoor interference, the multi-path effect, noise, and changeable channel conditions related to the dynamic indoor environment. Therefore, great care is required when designing an RSS-based target L&T algorithm to avoid high localization errors.

Probably the oldest and simplest RSS-based L&T system is trilateration [13,14]. In trilateration, RSSI measurements can be transformed directly into distances between the underlying transmitters and receivers. Thereafter, the target location can be estimated with the help of three minimum distances. However, the trilateration technique generally suffers from error uncertainty propagation and cannot handle environmental dynamicity effectively, leading to very poor localization accuracy. In complex indoor environments where the interference, reflection and refraction of signal forces are superimposed on specific points, fingerprint-based methods involving the use of machine learning (ML) can be superior to the trilateration method in terms of localization accuracy, since position estimation is based on a data matching algorithm using a set of reliable RSSI data selected from a prebuilt fingerprint database [15]. Among the data processing techniques, ML algorithms are the most promising. Traditional target localization methods locate the target in an iterative manner. However, the dynamicity in wireless signals generally produces localization error in the estimated target position. This error is then propagated and magnified rapidly in subsequent iterations. A significant advantage of going with a ML-based localization approach is that ML-based schemes for WSNs do not estimate target position in an iterative manner. Due to their adaptive nature to changing indoor conditions, ML techniques are deemed to be effective in eliminating the need for unnecessary redesign. In the offline phase, a target localization model is trained with a suitable dataset to learn the relationship between RSSI measurements and the corresponding reference positions. Once the proposed model is trained, random real-time RSSI measurements are fed to it as inputs to estimate the corresponding target location in the online phase. A support vector machine (SVM) is one of the important types of ML, which has higher data fitting capability, global optimality, and fewer control parameters [16,17,18]. Due to their remarkable generalization capability, SVMs have also gained preference in regression estimation problems (denoted support vector regression, SVR). Compared to popular ML models, such as the back propagation neural network (BPNN), the radial basis function (RBF) neural network, multilayer perceptron (MLP), and the generalized regression neural network (GRNN), SVR shows superior forecast performance [15]. The research objective of this work is to exploit the potential benefits of SVR to solve an indoor target localization problem. The research work in this paper was carried out in two phases. The key contributions of this work are:

(1) We proposed a novel SVR-based target localization framework to address the issues with RSS-based localization of a single target moving in an indoor environment. The proposed SVR-based scheme was compared through simulations with the classical trilateration technique and our previously published GRNN-based localization scheme in phase I. Unlike the GRNN-based scheme, the proposed SVR-based scheme needed only three RSSI measurements for target localization and still showed improved localization accuracy compared to that with GRNN and trilateration.
(2) Subsequently, the proposed SVR framework was fused with a standard KF to form yet another target localization framework, named SVR+KF. This proposed SVR+KF scheme was compared with respect to localization through simulations with the proposed SVR-based scheme and the classical trilateration scheme in phase II. The second proposed SVR+KF scheme yielded centimeter-level target localization accuracy.
(3) The target’s motion in both phase I and phase II was assumed to have a moving trajectory and high variation in velocity during motion. RSSI measurement noise and target motion statistics were kept the same in both phases. Simulation results in both phases demonstrated that the proposed SVR-based schemes effectively dealt with noisy RSS measurements and dynamic target motion, compared to trilateration and GRNN.

The manuscript is organized as follows. Section 2 discusses related recent works in RSS-based target L&T, followed by the introduction to the proposed SVR architecture for localization in Section 3. Section 4 presents the overall system design, whereas discussion on results obtained is presented in Section 5. At the end the key research findings are given in Section 6.

## 2. Related Work

Indoor localization methods exploiting RSSI field measurements can be classified into two principal branches: machine learning methods and filter-based methods. The first approach is basically a supervised learning-based approach for target L&T through RF fingerprinting. There are a number of possibilities with this first approach, such as KNN (k-Nearest Neighbour), radial basis function (RBF), multilayer perceptron (MLP), extreme learning machine (ELM), CNN (Convolutional Neural Network), recurrent Neural Network (RNN), back propagation neural network (BPNN), and SVM. These methods involve a training phase, in which mapping between RSSI measurements and corresponding target locations is conducted. Based on this analysis, the underlying supervised learning model parameters are updated for the given indoor RF environment. Once the underlying model is trained, in the offline phase, real-time target locations can be estimated for any random RSSI field measurements. RF fingerprinting using RSSI measurements for an indoor environment wherein the target moves was carried out in [19]. During the online location estimation phase, 𝑘-nearest positions were computed using the least squares method. By averaging these 𝑘-nearest positions, the target location was computed at the end. The authors in [20] proposed a kernel online sequential extreme learning machine (KOS-ELM) scheme incorporating RF fingerprinting and trilateration for target localization in the offline stage. The KNN framework was then used target localization during the online estimation phase. Wafa et al. [21] deployed a CNN-based localization framework for an Internet of Things (IoT) sensor system for target localization. In this work, the 2D localization problem was converted into a 3D tensor identification problem. The concept of a 3D image tensor constructed with a 2D matrix of RSSI measurements yielded average localization accuracy of 2 m. Yet another work based on CNN, adopting the concept of hybrid wireless fingerprint localization, was proposed by [22], utilizing a RSSI ratio for various access points (APs). Large numbers of RSSI fingerprints were obtained for an area of 12.5 m × 10 m from deployed APs for fifteen days. The average localization errors obtained with KNN-, SVM-, and CNN-based approaches were 4.1681 m, 4.1145 m, and 3.9118 m, respectively. Although CNN was superior to other methods, it relied on parameters such as learning rate, activation function, and threshold process. For high localization accuracy, these CNN parameters must be checked for every entry in the training database. This is a time-consuming task, and thus a CNN-based localization approach may be accurate for specific system conditions but is not suitable in general. The authors in [23] proposed a RSS-based robot indoor positioning scheme based on the kernel extreme learning machine (K-ELM) algorithm. The authors took 68,500 samples of RSSI measurements for a 32 m × 16 m area using eight APs. The proposed fingerprint-based localization scheme was evaluated using the proposed K-ELM scheme as well as Bayesian, KNN, classic ELM, and online sequential ELM (OS-ELM) algorithms. The proposed K-LEM-based scheme obtained localization accuracy of 8.125 m, which was superior to the other considered methods. The BPNN can also be utilized for indoor target localization [24]. However, the important limitation with BPNN is the need for multiple iterations for converging to the optimal location estimation.

Nguyen et al. [25] proposed a SVM-based localization scheme for target localization using ad hoc networks. The proposed scheme assumes that there is connectivity of all nodes with each other, and the positions of the anchor nodes in the network are known in advance. The classification model was obtained using field measurements collected by the anchor nodes, which were then utilized to find the target location during the online location estimation stage. However, the proposed SVM-based localization scheme worked well for networks with densely distributed sensors. Tran and Nguyen [26] analyzed a proposed SVM-based localization scheme for WSN-based target localization. In this work, the authors determined the upper bound of the localization error. Utilizing this upper bound, target localization accuracy was improved using an advanced optimization technique based on the concept of the mass spring. The authors in [16] proposed a multi-class SVM trained with RSSI field measurements for zoning localization. The proposed SVM-based framework was trained using datasets collected from two real world scenarios, namely, a laboratory building and a hospital. The proposed model yielded improved estimation accuracy compared to that with an ANN-based scheme. The authors in [27] proposed a hybrid indoor target localization model based on two kinds of measurements, namely, RSSI and channel state information (CSI). Initially, dimension reduction was achieved using principal component analysis (PCA) in the off-line stage by utilizing the CSI measurement. Subsequently, SVM was used to obtain the location-based regression function using the target locations that could be estimated with accuracy of approximately 1 m. The authors in [17] proposed RF-based beacon localization using an unmanned aerial vehicle (UAV) guided with the help of a pure pursuit guidance law. The proposed scheme, based on SVR, could directly locate the beacon by using RSSI measurements. The simulation result with the proposed SVR-based localization scheme yielded position accuracy within 2 m. In [8], the authors used a SVR-based target localization model with a RBF kernel. The authors investigated the localization efficacy of the proposed model by varying anchor density, link quality, and transmission power. Instead of treating target localization as a classification problem, the authors treated it as a regression problem. The authors in [18] proposed a least square support vector regression (LSSVR) localization scheme which used RSS-based ranging values as the inputs. To deal with fluctuation in the RSSI measurements, each time a new RSSI value was put into a queue, and the older value was removed from the queue. Each time, the average of all RSSI values was computed to ensure the stability of the queue over the sampling period of RSSI. During LSSVR-based localization, target localization error, the RBF kernel function parameter and the grid width parameter of LSSVR were optimized to improve target localization accuracy. In this work, the average localization error was computed by taking an average of all localization errors for the total length of the target trajectory. The obtained results demonstrated that the improvement in the average localization error of the proposed LSSVR algorithm without SVR parameter optimization was 21.82%, and with SVR parameter optimization was 11.70%.

In the filter-based localization approach, KF- and Particle Filter (PF)-based schemes are key techniques that have been utilized for a wide variety of target localization solutions. Being state estimation techniques, filter-based localization approaches involve two steps: prediction and updating (using real-time field measurements). The researchers in [28] proposed online semi-supervised support vector regression (OSS-SVR)-based target positioning, aiming to reduce the amount of labeled training data. Furthermore, the proposed algorithm was fused with KF and compared with semi-supervised manifold learning, an online Gaussian process and online semi-supervised localization. The simulation results proved that the OSS-SVR algorithm was robust to varying system noise and could estimate accurate locations using much less labeled training data. In [29] the authors implemented various ML techniques, such as recurrent neural network (RNN), multilayer perceptron (MLP), and radial basis function (RBF), and compared these with KF in the context of indoor target localization for a simulation area of 26 m × 26 m by deploying eight anchors on the area edges. The simulation results demonstrated that RBF outperformed the rest of the other techniques; however, MLP exhibited a better trade-off between computational complexity and localization accuracy. The experiment also concluded that KF demonstrated low average localization error but needed several iterations to produce low error compared with the rest of the architectures presented. We have previously fused GRNN with KF to design a robust localization system for moving targets in WSNs [30]. The proposed GRNN+KF and GRNN+UKF algorithms effectively dealt with the uncertainty in RSSI measurement noise. In these algorithms the GRNN architecture was trained with an input vector involving four RSSI measurements and corresponding 2-D locations of the mobile target. The GRNN based location estimates obtained were fed to KF or UKF to achieve improved location estimates compared to GRNN alone.

## 3. Proposed Support Vector Regression Model for Target Localization

**Figure 1:**
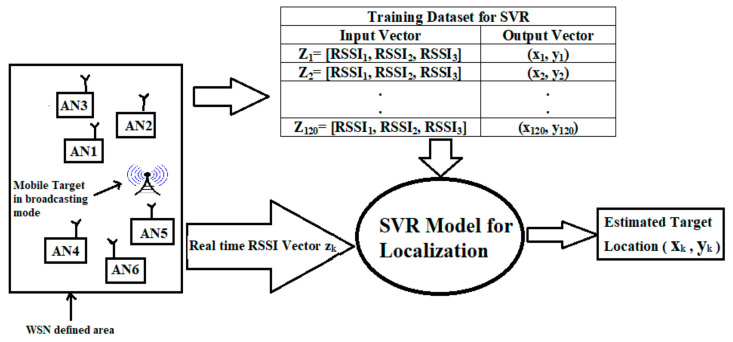
System block diagram for the proposed SVR-based target localization scheme.

ML methods based on kernel functions have been significantly successful in the context of tasks such as function approximation, classification, and regression analysis. Basically, SVM is a supervised ML model which can be utilized for support vector classification (SVC) and support vector regression (SVR). We mention here that we have not proposed a classification-based target localization model based on ML, due to various reasons. First, as compared to the case of regression, classification-based target localization schemes need more computational resources, especially for larger WSN areas. For instance, if we attempted to solve the indoor localization problem for an area of 100 m × 100 m, we would then need to break the total WSN area into 10 m × 10 m cells, resulting in twenty classes. Therefore, for a classification-based solution, it would necessitate the training of twenty separate ML models. Cell sizes smaller than 10 m × 10 m would require even greater numbers of classes, which in turn would demand more computational resources. Generally, localization solutions based on classification have a trade-off between efficiency and accuracy. By comparison, regression-based target localization schemes have no such trade-off. It is believed that if the data are transformed into high-dimensional feature space using SVM, classification ability will improve. SVR is capable of capturing nonlinear relationships in the feature space and therefore is also an effective tool for regression analysis. Additionally, SVR computational complexity does not rely on input space dimensionality. SVR has very good generalization ability and high data prediction accuracy. Due to the ability of SVR to work for problems of nonlinear forecast systems and identification systems, it can be applied for target localization and tracking. In the offline training stage, RSSI measurements collected from anchor nodes (ANs) deployed at predefined locations in the operational area and their corresponding target locations are stored together to form a training database. The proposed SVR localization model was trained with a set of 120 input vectors and the 120 corresponding 2-D locations of mobile targets for the considered WSN area in this work. During the online location estimation phase, any real-time RSSI measurements were applied to the trained SVR model. The SVR model then searched for similar RSSI patterns in the training database to find the closest possible match and return the closest possible target location corresponding to the given RSSI pattern. The proposed SVR model is graphically shown in Figure 1.

As we utilized RSSI measurements in the proposed scheme for WSN indoor target localization in our simulations, it is important to discuss the wireless channel model adopted to generate these RSSI values. Since the RSSI value is basically a received signal strength, which has variations among different radio chipsets, we preferred to use a more standardized logarithmic scale measure for RSSI values. On this logarithmic scale, a RSSI value closer to 0 dBm means a good quality received signal strength. In other words, we used a log-normal shadowing model (LNSM). RSSI measurements can be obtained via LNSM using Equation (1) [30,31]:

(1) $$z_{lj,k}=P_{r}(d_{0})-10n\log(d_{lj,k}/d_{0})+X_{\sigma },$$

where:

$\left(z_{lj,k}\right)$—RSSI measurement at node $N_{l}$ with coordinates $\left(x_{lk},y_{lk}\right)$ at time instance k. This RF signal is assumed to be transmitted by node $N_{j}$ with coordinates $\left(x_{jk},y_{jk}\right)$, 

$P_{r}(d_{0})$—RSSI at receiver kept at a distance $d_{0}$ (1 m), 

η—Path loss exponent,

$X_{\sigma }$—Normal random variable. 

The SVR model can be formulated using the concept of structural risk minimization, as given by Equation (2) [26]:

(2) $$F(z)=w^{T}z+b$$

where b and w are the SVR regression coefficients, and z is the RSSI vector. The optimal regression model for Equation (2) can be given by [31]:

(3) $$Minimize\frac{1}{2}∥w{∥}^{2}+C\sum_{i=1}^{N}(\xi_{i}+\xi_{i}{}^{*})\;\;subject\;to\left\{\begin{array}{c} F(z)-y_{i}\leq \varepsilon +\xi_{i}{}^{*}\\ y_{i}-F(z)\leq \varepsilon +\xi_{i}\\ \xi_{i},\xi_{i}{}^{*}\geq 0,\;i=1,2,....,N \end{array}\right\}$$

where

ε—Insensitive loss error function, 

C—Regularization factor, C>0, 

$\xi_{i}$, $\xi_{i}{}^{*}$—Slack variables representing upper and lower limitations on SVR. 

We used default values of C, γ, and ε, which were set to 1, 0.01, and 0.001, respectively. However, these parameters can be fine-tuned to obtain optimum results from the SVR model for the underlying application. In order to minimize Equation (2) subject to Equation (3), the regression function is given by Equation (4) [18,31]:

(4) $$f(z)=\sum_{i=1}^{N}(\alpha_{i}^{*}-\alpha_{i})K(z,z_{i})+B,$$

where 

$K(z,z_{i})$—Kernel function, 

B—bias value, and

$\alpha_{i}^{*},\alpha_{i}\geq 0$—Lagrange multipliers. 

Various types of kernel functions can be adopted with SVR [15,25]. In this work, we adopted the radial basis function (RBF) to create the SVR model because of its abilities of fast convergence, simplicity, and optimality in high-dimensional spaces, compared to other types of kernels [16]. The RBF kernel function is given by Equation (5):

(5) $$k(x,x_{i})=\exp\frac{\left(-\gamma ∥z-z_{i}{∥}^{2}\right)}{2\sigma^{2}}$$

where 

$∥z-z_{i}∥$—Euclidean distance between two points. These two points represent the value of an actual parameter of interest and the estimated parameter value for it.

σ—A parameter which defines a Gaussian function variance. It can be set manually and must be greater than zero.

## 4. System Design

**Figure 2:**
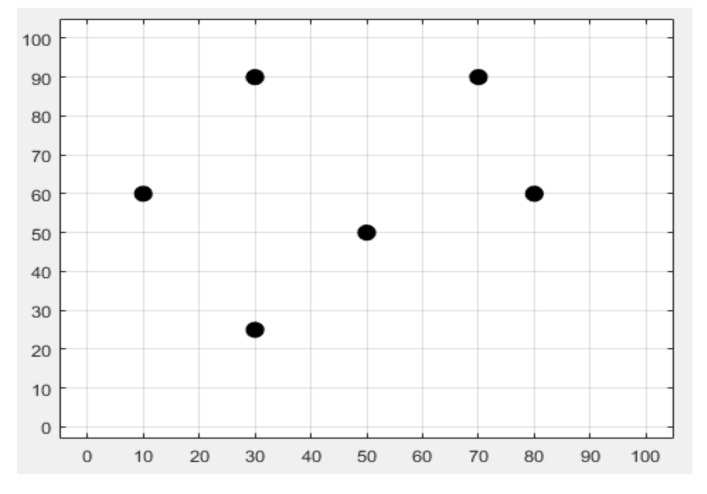
Anchor node deployment in the indoor environment.

This research work attempted to locate a single target moving in a 100 m × 100 m area with the help of only six anchor nodes in Phase I and Phase II, as shown in Figure 2. In the offline stage, the proposed SVR localization model was trained with a set of 120 input vectors and 120 corresponding 2-D locations of mobile targets for the considered WSN area in this work (See Figure 1). The same training dataset was utilized for Phase I and Phase II simulations. To provide a cost-effective solution, we used only six anchor nodes (ANs). We believe that if we wanted to track mobile targets for a larger area (i.e., more than 100 m × 100 m), we would require more than six ANs. During simulations six RSSI measurements were generated from the six ANs. These RSSI measurements were utilized as the input vector for the various localization schemes considered in this work. If the number of antennas (ANs) or even other system dynamics changed, it would be necessary to use a different training dataset. As discussed earlier, in phase I trilateration, GRNN, and the proposed SVR localization schemes were compared. In phase II the fusion of SVR and KF is proposed, and the proposed SVR and SVR+KF schemes were compared against traditional trilateration in the context of localization accuracy. All of the localization schemes considered in this work used RSSI measurements as inputs for estimating target locations. RSSI measurements closer to 0 dBm implied less distance between the AN and the target and better RF signal (RSSI) quality. Although six ANs were deployed in the WSN operating area, any three ANs re sufficient to effectively locate the mobile target with the proposed SVR and SVR+KF based target localization schemes. That means we considered RSSI measurements from AN1, AN2, and AN3 in both phase I and phase II. Each AN was presumed to have a transceiver for RF communication. The positions of six ANs were randomly determined in the considered 100 m × 100 m WSN area. The GRNN-based scheme considered RSSI measurements from AN1, AN2, AN3, and AN4, whereas the trilateration-based scheme considered RSSI measurements from all ANs, and utilized three RSSI measurements with high values (i.e., RSSI measurements from the three ANs which were closer to the target at a particular time instance). In case of trilateration implementation, we used the highest three RSSI measurements out of the six RSSI measurements obtained from the six ANs. For GRNN we used four RSSI measurements (from AN1 to AN4) as the input vector in implementation. However, for the proposed SVR and SVR+KF target localization schemes, we used only three RSSI measurements (from AN1 to AN3) as the input vector in implementation. We further clarify that we could have also used the other three RSSI measurements (from AN4 to AN6) as an input vector in implementation. Thus, the proposed SVR and SVR+KF localization schemes had fewer constraints with respect to RSSI measurements from ANs for location estimation, as compared to the trilateration- and GRNN- based schemes. The mobile target was assumed to carry a receiving node, which is supposed to receive RSSI measurements (RF signals) for each time step k during its motion from the six ANs deployed in the WSN area. The RSSI measurements from the six ANs are designated as RSSI_1_ to RSSI_6_ respectively. The deployment of ANs is given in Table 1 and shown in Figure 2. These ANs were randomly deployed in the given WSN area, and were presumed to be static. 

The target was assumed to take a total of 40 positions in the WSN area during its motion, and these were to be estimated with the help of trilateration or the proposed SVR and SVR+KF schemes. In the offline phase, the proposed SVR and SVR+KF architectures were trained using 120 sets of RSSI measurements and the 2-D locations corresponding to these RSSI measurements, as shown in Figure 1. Once the proposed SVR-based localization architecture was trained, it could be used to estimate mobile target location during the online localization stage. In the online phase, for each target position in the WSN area during its motion, the input vector ($Z_{k}$) for GRNN and SVR architectures at specific time instance k would be as given below in Equation (6) and Equation (7), respectively:

(6) $$Z_{k}=[RSSI_{1},RSSI_{2},RSSI_{3},RSSI_{4}],\;k=1,2,....,40$$

(7) $$Z_{k}=[RSSI_{1},RSSI_{2},RSSI_{3}],\;k=1,2,....,40$$

**Figure 3:**
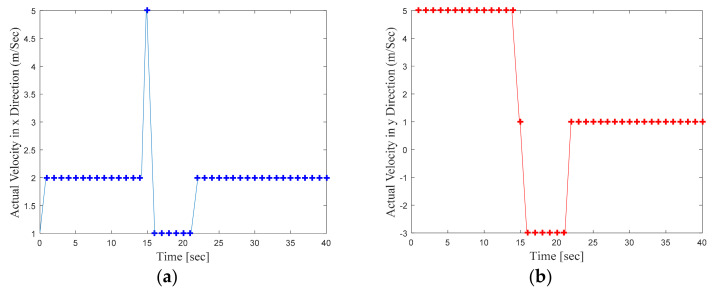
(**a**) Target velocity variation along x direction, (**b**) Target velocity variation along y direction.

The state vector for a mobile target at time instance k is $X_{k}=(x_{k},y_{k},{\dot{x}}_{k},{\dot{y}}_{k})’$. Here $x_{k}$ and $y_{k}$ specify the position, and ${\dot{x}}_{k}$ and ${\dot{y}}_{k}$ specify the speed in x and y directions respectively at the $k^{th}$ time instance, which are given by following equations.

(8) $$x_{k}=x_{k-1}+{\dot{x}}_{k}\;dt\;,$$

(9) $$y_{k}=y_{k-1}+{\dot{y}}_{k}\;dt\;,$$

where dt is the time period between two consecutive time instances such that dt=k−(k−1)  and is defined as 1 s. The abrupt changes in target velocity during the total target motion of T=40 s are defined by Equations (10)–(13). The changes in the mobile target velocity along x and y directions with respect to time are shown in Figure 3a, and Figure 3b respectively.

(10) $${\dot{x}}_{k}=2\;m/s,\;{\dot{y}}_{k}=5\;m/s,\;\;\;\;\mathrm{for}\;0<k<9\;\;s,$$

(11) $${\dot{x}}_{k}=5\;m/s,\;{\dot{y}}_{k}=2\;m/s,\;\;\;\;\mathrm{for}\;9\leq k\leq 15\;s,$$

(12) $${\dot{x}}_{k}=0\;m/s,\;{\dot{y}}_{k}=0\;m/s,\;\;\;\;\mathrm{for}\;16\leq k\leq 17\;s,$$

(13) $${\dot{x}}_{k}=2\;m/s,\;{\dot{y}}_{k}=-3\;m/s,\;\;\;\mathrm{for}\;18\leq k\leq 35\;s.$$

The efficacy in location estimation of the trilateration and proposed SVR and SVR+KF algorithms was obtained through three metrics, namely, average localization error, root mean square error (RMSE), and coefficient of correlation (R). For each k, we obtained the localization errors associated with x coordinate $\left({\hat{x}}_{k}-x_{k}\right)$ and y coordinate estimates $\left({\hat{y}}_{k}-y_{k}\right)$. The localization error for the $k^{th}$ time instance can be obtained by taking the average of these two error values. Subsequently, the average localization error during T can be determined using Equation (17). On similar lines, the RMSEs for the x and y coordinate estimates were computed first, and then by taking the average of these two RMSEs, we could obtain the average RMSE. For higher localization accuracy, localization error and RMSE must be as small as possible (ideally close to 0). R specifies the correlation strength between estimated and actual values. The R value must be close to 1 for high localization accuracy. The value of R was directly obtained from the MATLAB plotregression command.

(14) $$Average\;Localization\;Error=\frac{1}{T}\sum_{k=1}^{T}\frac{\left({\hat{x}}_{k}-x_{k})+({\hat{y}}_{k}-y_{k}\right)}{2}\;$$

where

$\left({\hat{x}}_{k},{\hat{y}}_{k}\right)$—Estimated target location for $k^{th}$ time instance,

$\left(x_{k},y_{k}\right)$—Actual target location at $k^{th}$ time instance.

(15) $$RMSE_{x}=\sqrt{\sum_{k=1}^{T}\frac{{\left({\hat{x}}_{k}-x_{k}\right)}^{2}}{T}}\;.$$

(16) $$RMSE_{y}=\sqrt{\sum_{k=1}^{T}\frac{{\left({\hat{y}}_{k}-y_{k}\right)}^{2}}{T}}\;.$$

(17) $$RMSE_{avg}=\frac{\left(RMSE_{x}+RMSE_{y}\right)}{2}$$

## 5. Discussion and Results

The idea behind conducting the simulation experiment in phase I was to explore the target localization capability of the proposed SVR-based target localization model as compared against that of trilateration- and GRNN-based schemes. As mentioned earlier in Section 3 and Section 4, trilateration exploited the advantage of all six ANs for localization for RSSI measurements, whereas GRNN and the proposed SVR relied on only four and three ANs respectively. Once it was confirmed that the SVR-based scheme outperformed the GRNN-based scheme, more focus was given to the SVR-based localization approach in phase II. The environmental and system setup for phase II was kept the same as that for phase I. The aim of phase II was to evaluate a SVR + KF based fusion scheme compared to the SVR-based scheme and the trilateration technique. As trilateration-based target localization using RSSI measurements is a widely used approach by the research community for evaluating proposed RSS-based algorithms, we maintained localization comparison with traditional trilateration in both phases. The important simulation parameters used in this study are provided in Table 2.

### 5.1. Phase I: Comparison of SVR with Trilateration and GRNN

**Figure 4:**
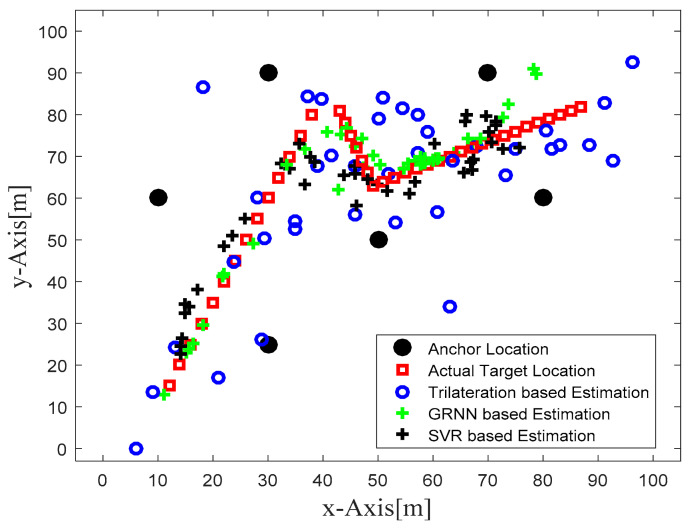
Phase I Result: Location estimation of the mobile target with trilateration-, GRNN-, and proposed SVR-based localization schemes.

**Figure 5:**
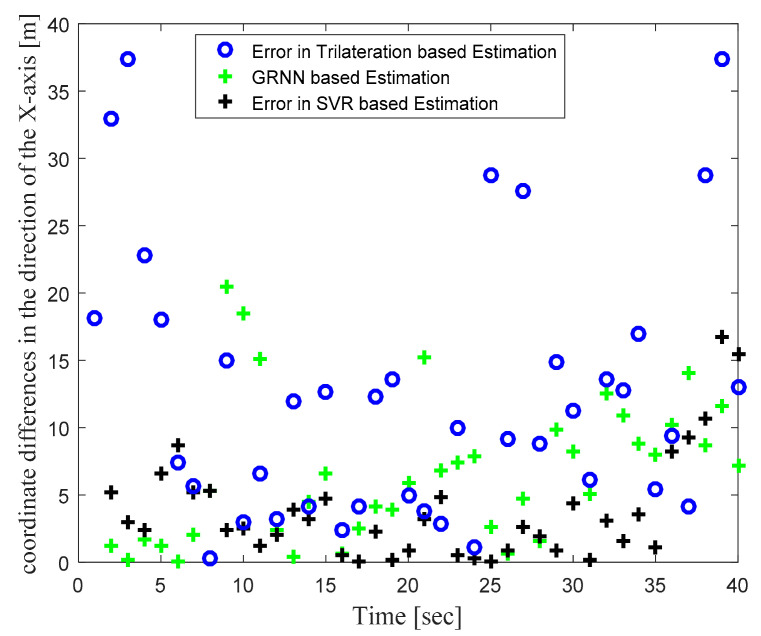
Phase I Result: Location estimation error in x direction with trilateration-, GRNN-, and proposed SVR-based localization.

**Figure 6:**
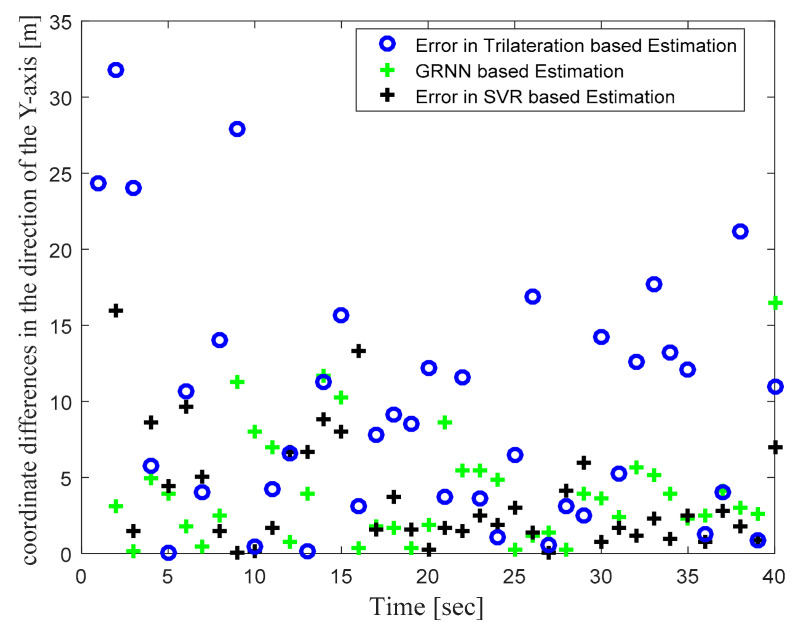
Phase I Result: Location estimation error in y direction with trilateration-, GRNN-, and proposed SVR-based localization.

**Figure 7:**
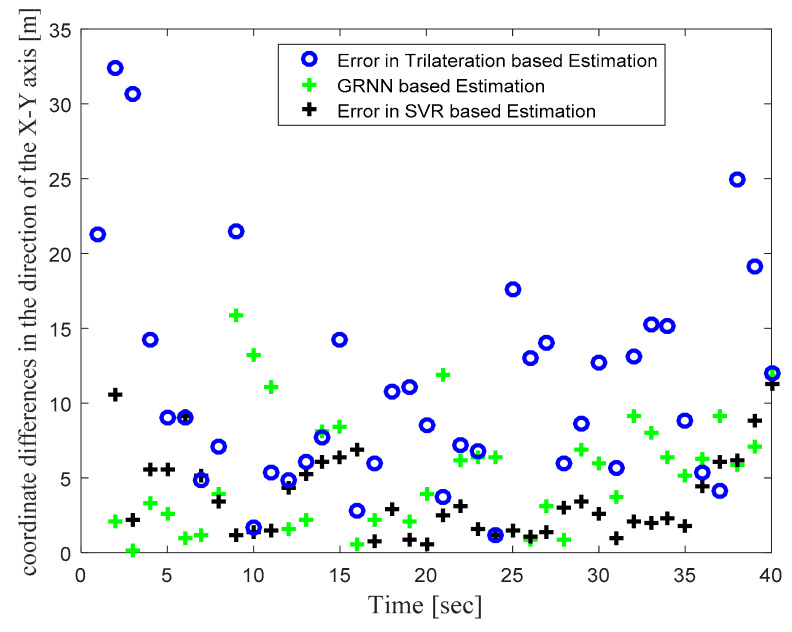
Phase I Result: Location estimation error in x–y direction with trilateration-, GRNN-, and proposed SVR-based localization.

Figure 4 illustrates the actual target track in the defined WSN area and the location estimates obtained with the trilateration-, GRNN-, and SVR-based localization schemes. Figure 4 clearly shows that the locations estimated with the proposed SVR-based scheme were closer to the actual target locations compared to those estimated with trilateration and GRNN. Although a few target location estimates obtained with SVR were away from the actual target locations, the location estimates obtained for those same target locations with trilateration and GRNN were further away from the actual target locations compared to the proposed SVR scheme. Figure 5 and Figure 6 plot the location estimation errors with the trilateration-, GRNN-, and SVR-based localization schemes in x direction and y direction respectively. In order to assess the overall estimation accuracy, the average of the estimation errors is plotted for each actual target location in Figure 7. From the results, it can be observed that location estimates obtained with the proposed SVR were far better than those obtained with trilateration and GRNN. From Figure 5, Figure 6 and Figure 7, it can be observed that estimation errors with the proposed SVR-based scheme were below approximately 15 m. The estimation errors with trilateration were the worst for many locations as compared to those of the other considered schemes. 

From Table 3 it is clear that the RMSE and average localization error with trilateration were very high as compared to those with GRNN- and SVR-based schemes. The average RMSE with GRNN and SVR decreased by 52% and 62% respectively compared to that with trilateration. The average localization error with GRNN and SVR decreased by 51% and 66% respectively compared to that with trilateration. In order to clarify the localization performance of the three considered localization schemes, four target locations were selected, and estimations obtained with the three considered schemes are compared in Table 3. For the first location (16, 25) considered in Table 4, we can see that estimations with GRNN were better than those with SVR, whereas the negative estimated coordinates with trilateration for the first location indicates that these were out of the defined WSN area considered during the simulation. In simple words, the estimations with trilateration for target location (16, 25) were outside the defined WSN area, which is why these are not visible in Figure 4. For other considered locations in Table 4, we can see that for some locations SVR yielded very close location estimates, while for others GRNN performed better.

**Figure 8:**
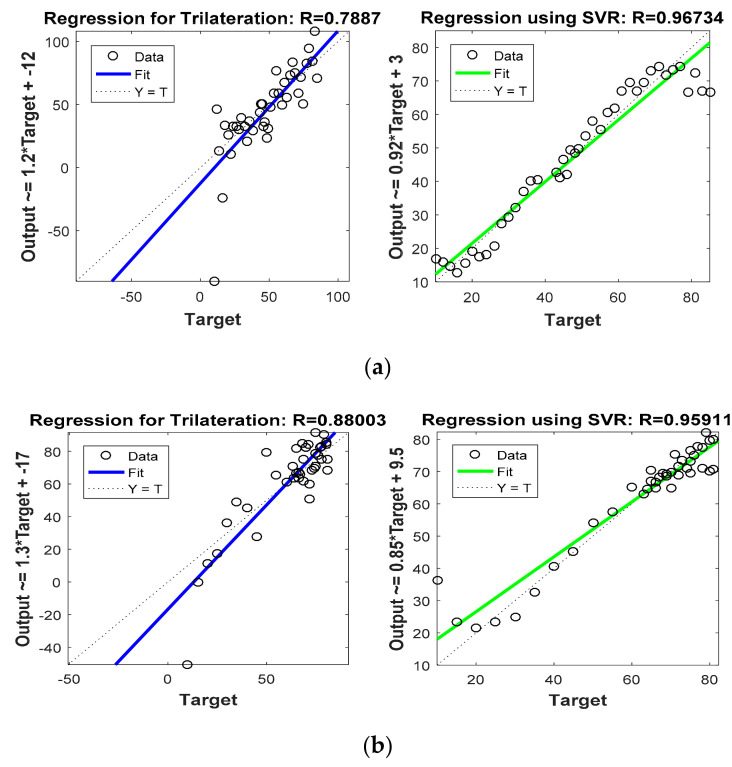
Phase I Result: (**a**) Regression coefficient with trilateration and proposed SVR for x direction, (**b**) Regression coefficient with trilateration and proposed SVR for y direction.

**Figure 9:**
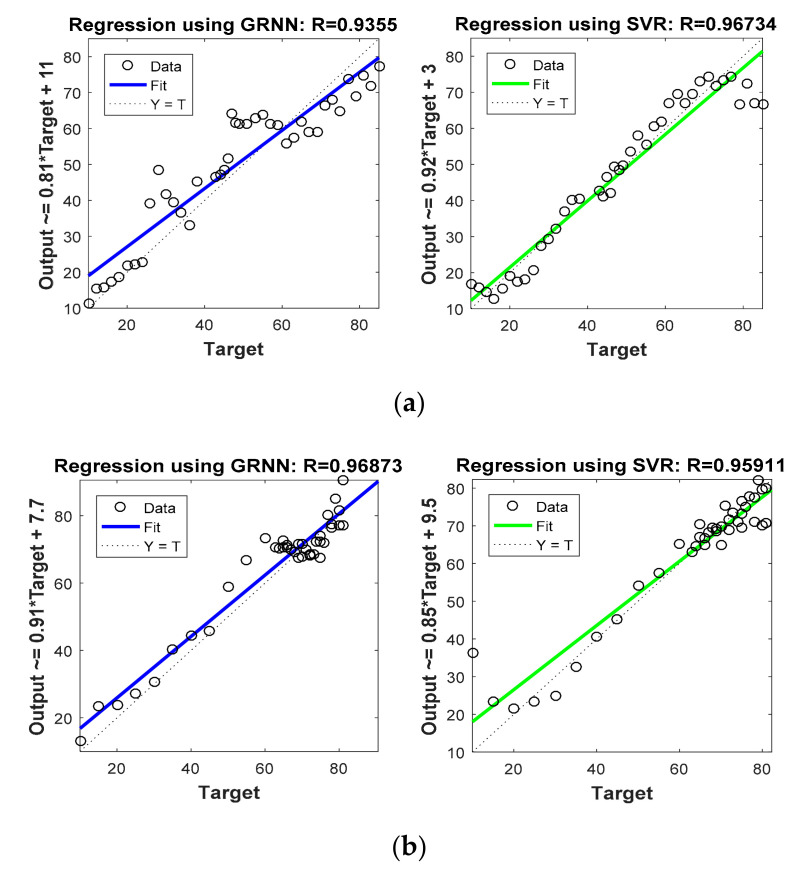
Phase I Result: (**a**) Regression coefficient with GRNN and proposed SVR for x direction, (**b**) Regression coefficient with GRNN and proposed SVR for y direction.

Figure 8 and Figure 9 are graphs which show correlations between actual target location (Target) and estimated target locations (Output). These figures compare the regression coefficients for the various localization schemes considered in Phase I. Figure 8a and Figure 9a show the regression coefficient analysis for x coordinate estimations, whereas Figure 8b and Figure 9b show the regression coefficient analysis for y coordinate estimations. For instance, the output expression on the y axis of first plot in Figure 8 was Output = 1.2 * Target + (−12). Comparing this equation with the standard equation of a line, we obtain the slope of the line in the plot at 1.2, and the y intercept at −12 (i.e., 12 positions down on the *y* axis). For more accurate estimation, the slope of the line in the plot must be close to 1. Thus, comparing this slope in the first half of Figure 8a (which is 1.2) with that in the second half of Figure 8a (which is 0.92), it is confirmed that SVR-based target tracking was superior to trilateration-based target tracking. From Figure 8 and Figure 9, and Table 5, it is clear that R values obtained for the SVR-based localization scheme were closer to 1 as compared to those obtained for trilateration and GRNN.

### 5.2. Phase II: Combination of SVR and Kalman Filter for Target Localization

**Figure 10:**
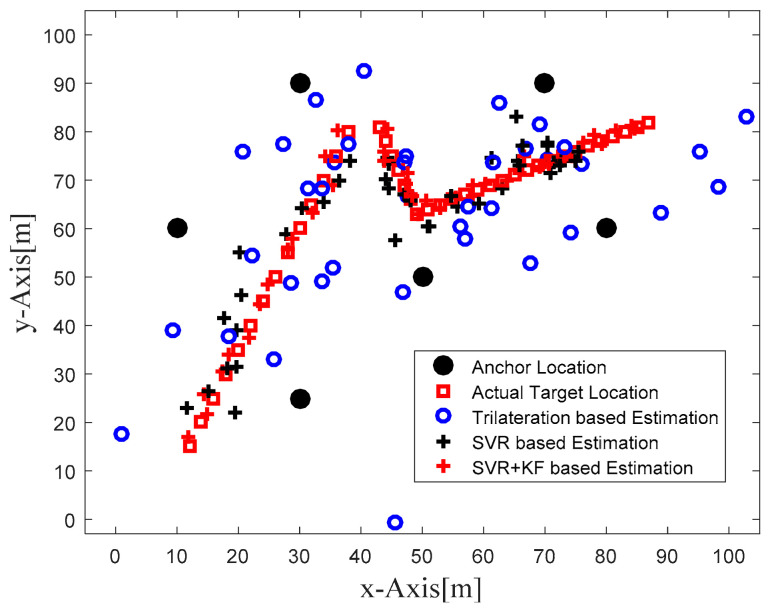
Phase II Result: Location estimation of mobile target with trilateration and proposed SVR- and SVR+KF-based localization.

**Figure 11:**
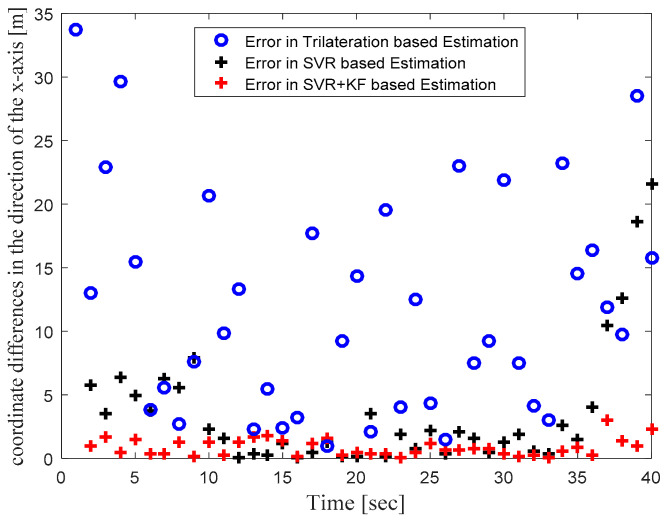
Phase II Result: Location estimation error in x direction with trilateration and proposed SVR- and SVR+KF-based localization.

**Figure 12:**
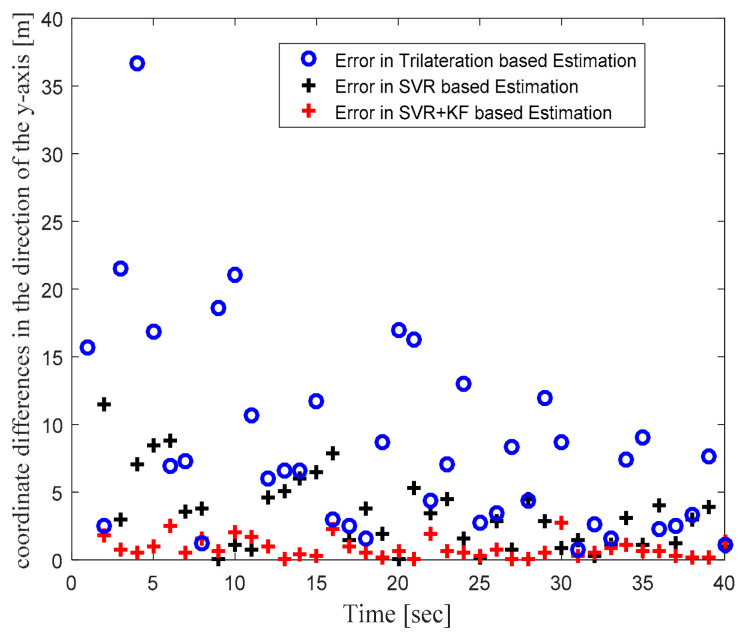
Phase II Result: Location estimation error in y direction with trilateration and proposed SVR- and SVR+KF-based localization.

**Figure 13:**
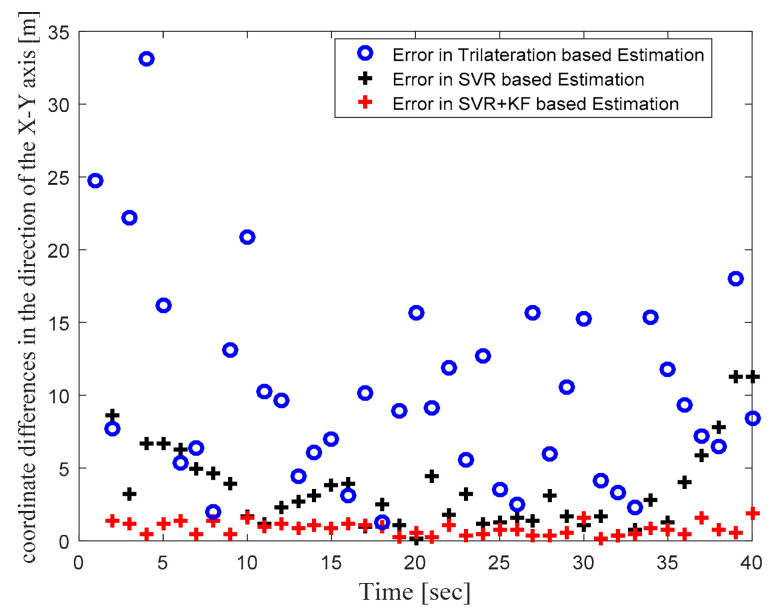
Phase II Result: Location estimation error in x–y direction with trilateration and proposed SVR- and SVR+KF-based localization.

**Figure 14:**
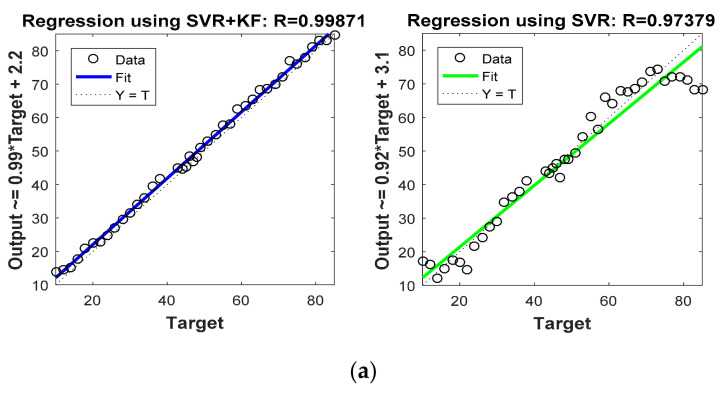

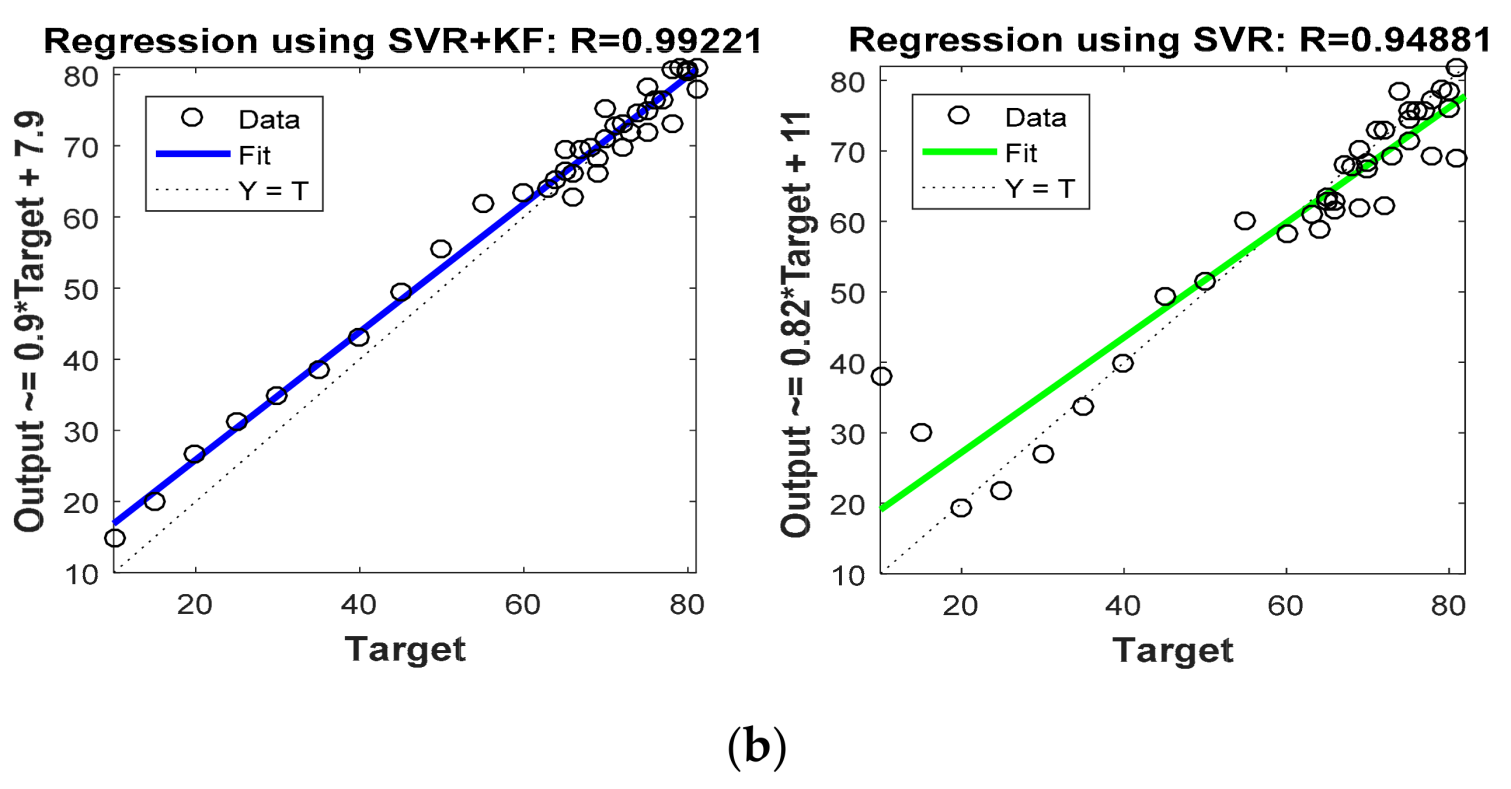
Phase II Result: (**a**) Regression coefficient with proposed SVR and SVR+KF for x direction, (**b**) Regression coefficient with proposed SVR and SVR+KF for y direction.

In order to focus more upon the SVR based scheme, in Phase II we compared SVR and SVR+KF based schemes with trilateration only. As in Figure 4 in Phase I, Figure 10 in Phase II illustrates the actual target trajectory and the estimates obtained with trilateration and both SVR-based localization schemes. From Figure 10 it is clear that SVR+KF-based estimations were even better than plain SVR-based estimations, and closely followed the actual target track. Figure 11 and Figure 12 plot the location estimation errors with trilateration and both SVR-based localization schemes in x direction and y direction, respectively. Figure 13 plots the average of estimation errors for each target location. From Figure 11, Figure 12 and Figure 13 it can be observed that the location estimation errors with the SVR+KF scheme were the lowest as compared to trilateration and plain SVR-based scheme, at well below 2.5 m. From Figure 13 it is observed that the estimation errors with trilateration were very high and varied from 2 to 26 m. Table 6 compares RMSE and average localization errors with the three considered localization schemes in Phase II. The average RMSE and average localization error with the SVR+KF scheme decreased by approximately 95% and 79%, respectively, compared to those with the plain SVR scheme. From Figure 14 and Table 7, it is seen that the R value with the SVR+KF scheme was very close to 1. 

In this work we also tested the time complexity of the trilateration, SVR, and SVR+KF localization schemes using MATLAB tic-toc commands. The time complexities of the trilateration, GRNN, SVR, and SVR+KF localization schemes were found to be 3 milli-sec, 3.2 milli-sec, 2.5 milli-sec, and 4 milli-sec respectively for considered system dynamics. That means that the proposed SVR-based scheme did not take much time to estimate mobile target locations. Thus, the fusion of SVR and KF yielded very high improvement in target localization accuracy. However, we believe that for different target trajectories or larger scales of the WSN area, such as 1000 m × 1000 m, the localization accuracy may vary. For a larger WSN operating area, one may need to deploy more ANs to track the mobile target. For such different system dynamics, one would need to increase and further customize the training dataset. However, once a custom RSS-based training dataset is generated, we believe that the proposed SVR-based localization schemes could yield better target localization performance.

## 6. Conclusions

This paper proposed a novel SVR-based target localization scheme to track a single target moving in an indoor environment with the help of RSSI field measurements. The proposed SVR-based scheme effectively dealt with highly fluctuating field measurements as well as high maneuvering in the target trajectory. For applications wherein a localization accuracy of 5 m to 6 m is required, the proposed plain SVR-based architecture would be a good lightweight option for indoor target localization. A target localization accuracy of approximately 5 m is sufficient for applications such as locating shopping carts in big shopping malls. In shopping malls, the locations of shopping carts equipped with wireless sensors can be very useful from a business point of view. This location information can be utilized to analyze customer behaviors systematically. For instance, from location information, the owner can analyze where customers spend more time and which sections of the shopping mall are most visited. Based on this, the placement of products may be changed to increase sales. By comparison, for applications demanding target tracking accuracy below 1 m, the proposed SVR+KF localization scheme would be a very good option. The results obtained from this research work provide a solid foundation for our future work, which aim to test the proposed learning model for various other SVR kernel functions such as linear, polynomial, sigmoid, etc. Another important future research direction that we would like to explore is to test the proposed scheme for larger WSN operating areas and multi-target tracking (MTT) in indoor environments. 

## Figures and Tables

**Table 1:** Deployment of anchor nodes in the simulations.

Anchor Node Number	2-D Location	Anchor Node Number	2-D Location
1	(30, 25)	4	(30, 90)
2	(10, 60)	5	(80, 60)
3	(50, 50)	6	(70, 90)

**Table 2:** Simulation parameters for Phase I and Phase II.

Parameter Name	Value
Initial Target State Vector *X*_0_ at *K* = 0	(12, 16, 0, 0)
AN communication radius	30 m
transmitter and receiver antenna gains	1 dB
Transmission power	1 mW
Discretization time step dt	1 s
Path Loss Exponent *η*	3
Normal Random Variable *X*_σ_	~N(3, 1)
RBF Kernel Function Constant σ	1

**Table 3:** RMSE and average localization error obtained in Phase I.

Name of Localization Algorithm	RMSE for x Coordinate	RMSE for y Coordinate	Average RMSE for x and y Coordinates	Average Localization Error
Trilateration	15.8403	12.7018	14.2711	11.0682
GRNN	7.6033	6.1926	6.8979	5.3772
SVR (Proposed)	5.0755	5.6407	5.3581	3.7995

**Table 4:** Location-wise estimation results for four target locations for Phase I.

Location Number	Actual Coordinate	Coordinates Estimated with Trilateration	Coordinates Estimated with GRNN	Coordinates Estimated with SVR (Proposed)
1	(16, 25)	(-0.70, -4.66)	(15.77, 24.15)	(19.24, 17.12)
2	(32. 65)	(23.74, 84.23)	(37.22, 73.01)	(33.67, 68.80)
27	(55, 66)	(41.76, 52.54)	(56.58, 68.10)	(55.77, 65.37)
35	(85, 81)	(86.99, 73.01)	(79.74, 96.21)	(70.77, 82.51)

**Table 5:** Comparison of R values for Phase I.

Name of Localization Algorithm	R Value for x Coordinate Estimation	R Value for y Coordinate Estimation
Trilateration	0.7887	0.88003
GRNN	0.9355	0.96873
SVR (Proposed)	0.96734	0.95911

**Table 6:** RMSE and average localization error obtained in Phase II.

Name of Localization Algorithm	RMSE for x Coordinate	RMSE for y Coordinate	Average RMSE for x and y Coordinates	Average Localization Error
Trilateration	13.6668	14.9266	14.2967	11.2034
SVR	5.6929	5.8932	5.7930	4.0430
SVR+KF (Proposed)	0.3497	0.1725	0.2611	0.8528

**Table 7:** Comparison of R values for Phase II.

Name of Localization Algorithm	R Value for x Coordinate Estimation	R Value for y Coordinate Estimation
SVR	0.97379	0.94881
SVR+KF (Proposed)	0.99871	0.99221

## Data Availability

This research has no datasets.
